# Association between albumin–bilirubin grade and plasma trough concentrations of regorafenib and its metabolites M-2 and M-5 at steady-state in Japanese patients

**DOI:** 10.1007/s10637-024-01429-z

**Published:** 2024-03-22

**Authors:** Kazuma Fujita, Daiki Taguchi, Koji Fukuda, Taichi Yoshida, Kazuhiro Shimazu, Hanae Shinozaki, Hiroyuki Shibata, Masatomo Miura

**Affiliations:** 1 Department of Pharmacy, Akita University Hospital, Akita, Japan; 2 Department of Clinical Oncology, Akita University, Akita, Japan; 3 Department of Pharmacokinetics, Akita University Graduate School of Medicine, 1-1-1 Hondo, Akita, 010-8543 Japan

**Keywords:** Regorafenib, M-2, M-5, Albumin–bilirubin grade, Overall survival, Plasma concentration

## Abstract

The aim of the present study was to determine whether the trough plasma concentrations (C_0_) of regorafenib and its metabolites, the N-oxide metabolite (M-2) and the desmethyl N-oxide metabolite (M-5), in 21 patients receiving regorafenib therapy were affected by albumin-bilirubin (ALBI) grade. Regorafenib was administered at dosages ranging from 40 to 160 mg once daily on a 3-week-on, 1-week-off cycle. C_0_ values of regorafenib and its major metabolites were measured by high-performance liquid chromatography on day 8 after treatment initiation. The C_0_ values of regorafenib and metabolites M-2 and M-5 were significantly lower in patients with ALBI grade 2 as compared with grade 1 (*P* = 0.023, 0.003 and 0.017, respectively). The total C_0_ of regorafenib and its metabolites was significantly higher in ALBI grade 1 patients relative to grade 2 (3.489 μg/mL vs. 1.48 μg/mL; *P* = 0.009). The median relative dose intensity (RDI) of patients categorized as ALBI grade 2 was significantly lower than that of grade 1 patients (21.9% vs. 62.9%; *P* = 0.006). In 15 colorectal cancer patients among the total 21 patients, patients with ALBI grade 2 (n = 9) had a significantly shorter median overall survival time than patients with grade 1 (n = 6; *P* = 0.013). Administering a low dose of regorafenib to patients with ALBI grade 2 reduces the RDI of regorafenib and lowers treatment efficacy, as an appropriate C_0_ of regorafenib is not maintained. Monitoring the C_0_ of regorafenib regularly is necessary to guide dose adjustment.

## Introduction

Regorafenib is an oral small-molecule tyrosine kinase inhibitor that targets several receptors, including vascular endothelial growth factor receptor 1–3, fibroblast growth factor receptor, platelet-derived growth factor receptor α, stem cell factor receptor and rearranged during transfection [1, 2]. It has been approved for the treatment of metastatic colorectal cancer (CRC), gastrointestinal stromal tumor (GIST) and hepatocellular carcinoma (HCC). Regorafenib is mainly metabolized by cytochrome P450 (CYP) 3A4 to an N-oxide metabolite (M-2) and a desmethyl N-oxide metabolite (M-5) [3]; however, the M-2 and M-5 metabolites exhibit similar activity against target kinases as does the parent compound [3].

Regorafenib therapy is associated with several important toxic effects. For example, in the CORRECT trial conducted in CRC patients, grade 3 or 4 severe adverse events, such as skin reactions of the hands or feet, occurred in 54% of patients receiving regorafenib therapy [4]. Accordingly, relationships of the plasma concentrations of regorafenib and its metabolites M-2 and M-5 with the efficacy and toxicity of the drug have been intensively studied [5–9]. In particular, these concentrations can influence the adverse effects that often necessitate dosage reductions or even discontinuation of treatment. Plasma concentrations of M-5 in particular have been reported to be significantly correlated with the onset of skin toxicities [5, 6].

The albumin-bilirubin (ALBI) grade was initially developed as a measure of liver function in patients diagnosed with HCC [10]. This measure has been shown to have predictive power in multiple contexts; for example, a high ALBI grade is a predictor of poor prognosis in patients with HCC who are undergoing treatment with lenvatinib or sorafenib [11–13]. Similar to the results in HCC patients, the ALBI grade has been reported to be significantly correlated with overall survival with regorafenib therapy in CRC patients [14]. However, no clinical studies have investigated the association between ALBI grade and the plasma concentrations of regorafenib in cancer patients receiving regorafenib therapy. Specifically, regorafenib is known to increase bilirubin levels by inhibiting the bilirubin metabolic enzyme uridine diphosphate glucuronosyltransferase (UGT) 1A1 [15]. Despite these intriguing connections, the effects of *UGT1A1* polymorphisms on the relationships between ALBI grade and regorafenib therapy have not yet been studied.

Therefore, the aim of the present study was to determine whether the plasma concentrations of regorafenib and its metabolites M-2 and M-5 in 21 patients receiving regorafenib therapy were affected by liver function as indicated by ALBI grade.

## Methods

### Patients and protocols

Twenty-one Japanese patients with CRC, HCC or GIST who were taking regorafenib (STIVARGA®; BAYER Co., Ltd, OSAKA, Japan) and were treated at the Akita University Hospital from June 2014 through September 2021, were consecutively enrolled in the study. The inclusion criteria were in accordance with standard eligibility criteria for regorafenib treatment [16]. The study was conducted according to the principles of the Declaration of Helsinki. The study protocol was approved by the Ethics Committee of Ito Hospital and Akita University School of Medicine (nos. 790 and 2826), and all patients provided written informed consent for participation in the study.

Regorafenib was orally administered at dosages ranging from 40 to 160 mg once daily on a 3-week-on, 1-week-off cycle. The initial dosing was determined by clinicians and was adjusted according to patient age, Eastern Cooperative Oncology-Group (ECOG) performance status, nutritional status, chemotherapy line and the severity of adverse events during previous therapies. Dose reductions of regorafenib were carried out based on the grade of each side effect. During the therapy period, patients were instructed to not consume or ingest drugs or foods that are known to affect the function of CYP3A or P-glycoprotein. The trough plasma concentration (C_0_) of regorafenib at the steady-state was determined from samples drawn on day 8 following the initiation of regorafenib administration. Plasma was isolated from whole blood by centrifugation at 1900 × *g* for 15 min and was stored at -40 °C until analysis.

### ALBI score

ALBI scores were calculated with the following formula: ALBI score = (log_10_ bilirubin [μmol/L] × 0.66) + (albumin [g/L] ×  − 0.085). Patients were stratified into three grades based on ALBI score, as reported previously [10]: grade 1, score ≤  − 2.60; grade 2, − 2.60 < score ≤  − 1.39; and grade 3, score >  − 1.39. In the present study, there were no patients classified as ALBI grade 3; therefore, the patients were divided into an ALBI grade 1 group and an ALBI grade 2 group, and comparisons were made of patient characteristics, C_0_ values and dose-adjusted C_0_ values of regorafenib, M-2 and M-5 between the two groups.

### Analytical methods

Plasma concentrations of regorafenib were measured by high-performance liquid chromatography (HPLC) as described previously [17]. Briefly, sorafenib (100 ng in 10 µL methanol) was added as an internal standard to 100-µL of plasma. This sample was diluted with 900 µL water and vortexed for 30 s. This mixture was applied to an Oasis HLB extraction cartridge that had been activated previously with methanol and water (1.0 mL each). The cartridge was then washed with 1.0 mL water and 1.0 mL of 20% methanol in water and eluted with 1.0 mL of 100% methanol. Eluates were dried by vortex-vacuum evaporation at 60 °C using a rotary evaporator (AS-ONE CVE-2AS; Osaka, Japan).

The resulting residue was dissolved in 20 µL methanol and vortexed for 30 s. The sample was then diluted with 20 µL of the mobile phase and then vortexed for an additional 30 s. A 20-µL aliquot of the sample was processed by HPLC. The HPLC system comprised a PU-2080 Plus chromatography pump (JASCO, Tokyo, Japan) equipped with a CAPCELL PAK C18 MG П HPLC column (250 mm × 4.6 mm I.D.; Shiseido, Tokyo, Japan), a UV-2075 light source and an ultraviolet detector (JASCO). The mobile phase was 0.5% KH_2_PO_4_ (pH 3.5):acetonitrile (30:70, v/v), which was degassed in an ultrasonic bath prior to use. The column was developed at ambient temperature with a flow rate of 0.5 mL/min, and sample detection was carried out at 260 nm. Coefficients of variation for intra-and inter-day assays were less than 12.2% for regorafenib, less than 12.3% for M-2 and less than 15.1% for M-5. The lower limits of quantification for regorafenib, M-2 and M-5 were 10 ng/mL for each analyte.

### Identification of genotypes

DNA was extracted from peripheral blood samples using a QIAamp Blood Mini Kit (Qiagen, Tokyo, Japan) and was stored at -80 °C until analysis. The alleles of *UGT1A1* were genotyped using a fully automated single nucleotide polymorphism (SNP) detection system (Prototype of i-densy, ARKRAY Inc., Kyoto, Japan). The following variant sequences of *UGT1A1* were studied: a 2-nucleotide insertion (TA) within the TATA box resulting in the sequence (TA)7TAA (^1^39 to ^1^53, UGT1A1*28: rs8175347); and a transition (+ 211 from the initial site of the transcription, G to A) at codon 71 in exon 1 that changes glycine to arginine (G71R, UGT1A1*6: rs4148323).

### Statistical analyses

The Kolmogorov–Smirnov test was applied to assess the distribution in each data set. Each patient characteristic value is presented as a median (range). Associations between ALBI grade and *UGT1A1* genotype frequency or patient characteristics were determined using the Pearson χ^2^ test. Relative dose intensity (RDI) values were calculated according to the following formula: RDI = (dose × number of days taken) × number of cycles / 160 mg × 21 d × number of cycles. Each C_0_ and dose-adjusted C_0_ of regorafenib, M-2, and M-5 for each ALBI grade and for each patient characteristic was expressed as the median (minimum–maximum values). Kruskal–Wallis tests or Mann–Whitney U tests were used to determine the differences between groups. The Spearman’s rank correlation coefficient test was used to assess correlations in continuous values between groups, and all results of this test were expressed as correlation coefficients (*r*). Survival curves for overall survival were analyzed using the Kaplan–Meier method. Differences or correlations with *P* values of less than 0.05 were considered statistically significant. Statistical analysis was performed using SPSS 26.0 for Windows (SPSS IBM Japan Inc., Tokyo, Japan).

## Results

The baseline characteristics of 21 patients with CRC, HCC or GIST who were receiving regorafenib therapy and were enrolled in the study are shown in Table 1. Nine of the patients (42.8%) were classified as ALBI grade 1, and twelve patients (57.2%) were classified as ALBI grade 2. The age of patients classified as ALBI grade 2 was significantly higher than that of patients classified as ALBI grade 1 (median 68.5 and 55.0 years, respectively). One of the patients classified as ALBI grade 1 received a regorafenib dose of 80 mg, five ALBI grade 1 patients were administered 120 mg regorafenib and three ALBI grade 1 patients were administered 160 mg regorafenib. Three ALBI grade 2 patients received 40 mg regorafenib, six received 80 mg, two received 120 mg and one received 160 mg. The regorafenib dose was determined to be significantly different between the two groups (*P* = 0.035). The median RDI value of patients classified as ALBI grade 2 was significantly lower than that of patients classified as ALBI grade 1 (21.9% vs. 62.9%; *P* = 0.006).

**Table 1:** Characteristics of patients treated with regorafenib and categorized as ALBI grade 1 or ALBI grade 2

	ALBI grade 1						ALBI grade 2						*P* value
Patient number	9						12						
Female:male	4:5						3:9						0.397
Age, years (range)	55.0	(	43	-	70	)	68.5	(	43	-	83	)	0.015*
Body weight (kg)	55.0	(	41.2	-	75.8	)	56.9	(	44.2	-	85.4	)	0.702
Regorafenib initial dose (mg)													0.035*
40:80:120:160	0:1:5:3						3:6:2:1						
Tumor type													0.916
CRC:GIST:HCC	6:2:1						9:2:1						
ECOG performance status													0.486
0/1:2	9:0						10:2						
Number of metastatic sites													0.121
1:2: ≥ 3	1:5:3						6:5:1						
*UGT1A1* phenotype													0.911
NM:IM:PM	4:3:2						5:5:2						
Relative dose intensity (%)	62.9	(	14.3	-	74.3	)	21.9	(	12.5	-	55.9	)	0.006*
Laboratory test values at baseline													
Serum creatinine (mg/dL)	0.67	(	0.44	-	0.85	)	0.67	(	0.43	-	0.95	)	0.754
Aspartate transaminase (IU/L)	23	(	14	-	36	)	33	(	14	-	51	)	0.169
Alanine transaminase (IU/L)	18	(	10	-	34	)	19	(	13	-	58	)	0.345
Serum albumin (g/dL)	4.2	(	4.0	-	4.5	)	3.3	(	2.8	-	3.9	)	< 0.001*
Total bilirubin (mg/dL)	0.6	(	0.3	-	1.0	)	0.8	(	0.3	-	1.2	)	0.464

Variables are presented as number or median (minimum—maximum)

^*^Statistically significant between patients with ALBI grade 1 and ALBI grade 2

CRC: metastatic colorectal cancer; GIST: gastrointestinal stromal tumor; HCC: hepatocellular carcinoma

NM: normal metabolizer (UGT1A1*1/*1); IM: intermediate metabolizer (UGT1A1*1/*6 + *1/*28); PM: poor metabolizer (UGT1A1*6/*6 + *6/*28 + *28/*28)

Comparisons of C_0_ values of regorafenib, M-2 and M-5 with demographic and clinical characteristics of patients are shown in Table 2. The C_0_ of regorafenib was significantly correlated with age, regorafenib dose and aspartate transaminase (*r* = -0.436, 0.620 and -0.482; *P* = 0.035, 0.003 and 0.027, respectively). The C_0_ of M-2 was significantly correlated with age, aspartate transaminase, serum albumin and ALBI score (*r* = -0.488, -0.493, 0.532 and -0.452; *P* = 0.025, 0.023, 0.013 and 0.040, respectively). The C_0_ of M-5 was significantly correlated with age, regorafenib dose and aspartate transaminase (*r* = -0.466, 0.506 and -0.544; *P* = 0.044, 0.027 and 0.016, respectively). On the other hand, there were no significant differences between C_0_ of regorafenib, M-2 or M-5 and *UGT1A1* genotype groups (Table 2).

**Table 2:** Comparisons of clinical characteristics to the C_0_ values of regorafenib and its major metabolites (M-2 and M-5)

Genotype	n	Regorafenib - C_0_ (ng/mL)		*P* value	M-2 - C_0_ (ng/mL)		*P* value	M-5 - C_0_ (ng/mL)		*P* value
		Median	Minimum - Maximum		Median	Minimum - Maximum		Median	Minimum - Maximum	
*UGT1A1* genotype groups				0.600^b^			0.205^b^			0.258^b^
*1/*1	9	1371	687 - 3053		489	98 - 2997		252	38 - 2808	
*1/*6 + *1/*28	8	1021	405 - 1907		176	110 - 778		125	70 - 804	
*6/*6 + *6/*28 + *28/*28	4	1705	305 - 3649		912	186 - 3398		941	104 - 3796	
Gender				0.799^a^			0.224^a^			0.124^a^
Female	7	1311	405 - 3649		576	110 - 3398		544	125 - 3796	
Male	14	1319	405 - 3053		412	98 - 2997		134	38 - 2808	
ECOG performance status				0.400^a^			0.686^a^			0.070^a^
0/1	19	1386	405 - 3649		423	98 - 3398		254	38 - 3796	
2	2	956	890 - 1021		288	140 - 435		86	70 - 102	

		Correlation coefficient (*r*)		*P* value	Correlation coefficient (*r*)		*P* value	Correlation coefficient (*r*)		*P* value
Age (years)		-0.436		0.035*	-0.488		0.025*	-0.466		0.044*
Body weight (kg)		-0.073		0.753	-0.149		0.520	-0.159		0.515
Initial regorafenib dose (mg)		0.620		0.003*	0.340		0.131	0.506		0.027*
Laboratory test values at regorabenib initiation										
Aspartate transaminase		-0.482		0.027*	-0.493		0.023*	-0.544		0.016*
Alanine transaminase		-0.318		0.160	-0.358		0.111	-0.420		0.074
Serum albumin		0.364		0.104	0.532		0.013*	0.321		0.180
Total bilirubin		-0.055		0.812	0.277		0.224	0.321		0.180
Serum creatinine		0.005		0.982	0.057		0.805	-0.017		0.945
ALBI-score		-0.374		0.095	-0.452		0.040*	-0.339		0.156

Comparisons of ALBI grade with the C_0_ and dose-adjusted C_0_ values of regorafenib, of M-2, of M-5 and of the sum of regorafenib, M-2 and M-5 are shown in Table 3. The median C_0_ values of regorafenib, M-2 and M-5 and the median total C_0_ value in patients classified as ALBI grade 2 (regorafenib, 988 ng/mL; M-2, 297 ng/mL; M-5, 125 ng/mL; and total, 1480 ng/mL) were significantly lower than those in patients classified as ALBI grade 1 (regorafenib, 1907 ng/mL; M-2, 843 ng/mL; M-5, 799 ng/mL; and total, 3489 ng/mL) (Table 3). In addition, the median dose-adjusted C_0_ values of M-2 were significantly lower in patients classified as ALBI grade 2 than in those classified as ALBI grade 1 (5.0 ng/mL/mg vs. 8.8 ng/mL/mg, *P* = 0.023).

**Table 3:** Comparison of C_0_ and dose-adjusted C_0_ values for regorafenib and its major metabolites (M-2 and M-5) between patients classified as ALBI grade 1 and ALBI grade 2

	ALBI grade 1		ALBI grade 2		*P* value
	Median	Minimum—Maximum	Median	Minimum—Maximum	
Regorafenib					
C_0_ (ng/mL)	1907	686—3649	988	405—2258	0.023*
C_0_/dose (ng/mL/mg)	12.6	5.7—25.4	15.0	5.1—27.4	0.508
M-2					
C_0_ (ng/mL)	843	160—3398	297	98—481	0.003*
C_0_/dose (ng/mL/mg)	8.8	1.3—21.2	5.0	1.2—9.5	0.023*
M-5					
C_0_ (ng/mL)	799	80—3796	125	38—544	0.017*
C_0_/dose (ng/mL/mg)	6.7	0.7—23.7	2.4	0.5—6.8	0.156
Regorafenib + M-2 + M-5					
C_0_ (ng/mL)	3489	1004—10,843	1480	695—2913	0.009*
C_0_/dose (ng/mL/mg)	30.1	8.4—67.8	21.7	9.4—39.6	0.126

^*^Statistically significant

**Fig. 1:**
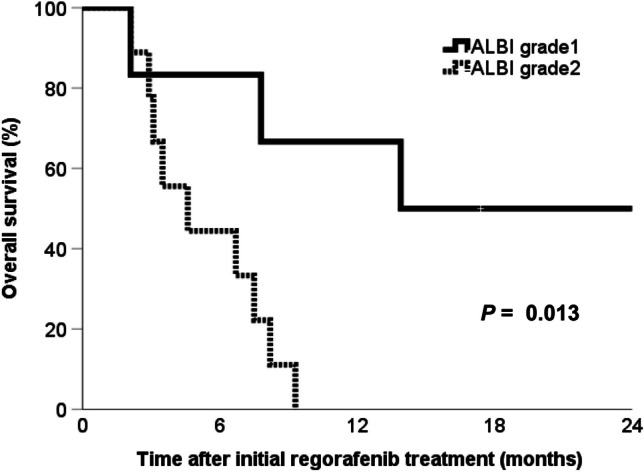
Overall survival curves of patients with metastatic colorectal cancer according to ALBI grade 1 or ALBI grade 2

Fifteen of the twenty-one study subjects were patients with CRC. Kaplan–Meier curves for the overall survival of the 6 CRC patients classified as ALBI grade 1 and the 9 CRC patients classified as ALBI grade 2 are shown in Fig. 1. The median overall survival upon regorafenib treatment in CRC patients classified as ALBI grade 1 was significantly longer than that in patients classified as ALBI grade 2 (13.9 months [95% confidence interval (CI), inestimable] vs. 4.6 months [95% CI, 1.4—7.8 months], *P* = 0.013).

## Discussion

To the best of our knowledge, this is the first report of an association between ALBI grade and plasma concentrations of regorafenib. The C_0_ values of regorafenib, M-2 and M-5 on day 8 after initiation of therapy in patients classified as ALBI grade 2 were significantly lower than the values in patients classified as ALBI grade 1. In addition, patients with ALBI grade 2 were older and had a poorer nutritional status, and they received a lower regorafenib dose with a lower RDI than did patients with ALBI grade 1. CRC patients classified as ALBI grade 2 also had a significantly shorter median overall survival time than did patients classified as ALBI grade 1. The results obtained in the present study provide evidence that ALBI grade might impact the efficacy and adverse effects of regorafenib treatment.

Consistent with our results, Watanabe et al. reported a significantly shorter overall survival time and time to treatment failure for patients classified as ALBI grade 2 or 3 as compared to those classified as ALBI grade 1 in CRC patients receiving regorafenib therapy, and an ALBI grade of 2 or 3 was able to predict poor treatment outcomes in these patients [14]. In that study, the plasma concentrations of regorafenib or of its metabolites were not determined; however, there was no significant difference in the median RDI between patients classified as ALBI grade 1 or ALBI grade 2 or 3 [14]. The dose of regorafenib in the inter-patients has previously been reported to not strongly correlate with plasma concentrations of the drug [2, 6, 9]. Therefore, the finding of variations in plasma concentrations among patients receiving the same dose was not unexpected. The efficacy and safety of treatment with regorafenib has been shown to be correlated with C_0_ values of the drug and its metabolites. For example, Fukudo et al. reported that the median progression-free survival time was significantly longer in patients achieving a total C_0_ value, including regorafenib and its two main metabolites, above 2.9 μg/mL than in those who did not [9]. They suggest that dose adjustments of regorafenib to achieve a total C_0_ between 2.9 and 4.3 μg/mL may improve the efficacy and safety of regorafenib [9]. Furthermore, in the TEXCAN phase II GERCOR trial, the overall survival time and safety associated with regorafenib therapy were reported to be favorable when the total C_0_ ranged between 2.5 and 5.5 μg/mL [18]. In the present study, the median total C_0_ of regorafenib and its metabolites in patients classified as ALBI grade 1 was determined to be 3.5 μg/mL; this value is within the target plasma concentration ranges suggested by these previous studies [9, 18]. Conversely, the total C_0_ of regorafenib and its metabolites in patients classified as ALBI grade 2 (1.48 μg/mL) was determined to be below these concentration ranges [9, 18]. Thus, the lower overall total C_0_ in the ALBI grade 2 group may contribute to the shorter overall survival of these patients in this context. In addition, due to association of the score with lower plasma concentrations, higher ALBI grades may be seen as a risk factor for poor prognosis in patients receiving regorafenib.

With regard to potential mechanisms governing associations between ALBI grade and regorafenib total C_0_ values, an *in vitro* study showed that serum albumin is the primary regorafenib-binding protein, with human plasma protein binding rates of 99.5%, 99.8%, and 99.95% for regorafenib, M-2, and M-5 respectively [19]. This finding would predict that the plasma concentrations of regorafenib and its metabolites in patients with ALBI grade 2 or 3, who have lower albumin levels, would be higher than those with ALBI grade 1, in contrast to our findings. However, in another study, the unbound fractions of regorafenib, M-2, and M-5 were reported to not be associated with serum albumin levels [8]. In addition, Pang et al. reported that the extent of protein binding by regorafenib was not affected by the level of physiologically available albumin [20]. Therefore, as plasma concentrations of regorafenib seem to not be influenced by serum albumin levels, additional research is necessary in order to determine the mechanisms through which the ALBI grade correlates with regorafenib pharmacokinetics.

Both C_0_ of regorafenib and its two metabolites were negative correlated with age and aspartate transaminase values. These data showed that regorafenib therapy typically involved lower daily doses for older patients and patients with higher aspartate transaminase values. However, only C_0_ of M-2 was found to be significantly associated with albumin levels. As a result of this, the C_0_ of M-2 adjusted by dose of regorafenib seems to be also significantly higher in patients with ALBI grade 1, who have higher albumin levels, as compared to those with ALBI grade 2. We cannot explain the reason why C_0_ of M-2 was associated with albumin levels. Similar to the parent drug regorafenib [8, 20], plasma concentrations of M-2 may not be influenced by serum albumin levels. Therefore, further study is necessary.

Another complicating factor in this study involves the potential role of hepatic dysfunction in the pharmacokinetics of regorafenib. Notably, the disposition pathway of regorafenib has been reported to involve the enterohepatic circulation [21, 22]. Namely, M-7 glucuronide is hydrolysed into regorafenib in the intestines, where the drug is subsequently reabsorbed. Generally, the clearance of glucuronide metabolites has been found to be significantly decreased in patients with poor renal function, and enterohepatic recirculation tends to be enhanced in these patients so as to compensate for the reduced renal activity [23]. Therefore, this observation suggests that the flux of regorafenib and its metabolites through the enterohepatic circulation is not expected to change due to hepatic dysfunction, because glucuronide metabolites are excreted through the kidney. The importance of renal excretion also suggests that reduced elimination of regorafenib in patients with severe hepatic dysfunction is likely due to decreased metabolic activity rather than by decreased enterohepatic circulation. However, in the present study, patients with hepatic dysfunction were not included, and additional research is necessary to clarify these relationships.

UGT1A1 catalyzes the glucuronidation of bilirubin, and polymorphisms of the *UGT1A1* gene have been reported to influence bilirubin metabolism and clearance [24]. However, in the present study, no significant difference in the frequency of *UGT1A1* genotype between ALBI grade 1 and grade 2 was identified. Notably, regorafenib inhibits the metabolism of bilirubin by UGT1A1, thereby increasing bilirubin levels [15]. Therefore, patients with *UGT1A1* poor metabolizer (PM) genotypes (*6/*6, *6/*28 and *28/*28) may be at elevated risk of regorafenib-induced hyperbilirubinemia as compared with patients with the UGT1A1*1 allele. The dose of regorafenib administered during the maintenance phase for patients with *UGT1A1* PM genotype may be lower than that for patients with the UGT1A1*1 allele. In the present study, however, no significant differences were identified in the RDI of regorafenib among groups with three different *UGT1A1* phenotypes (median values: 24.4% for normal metabolizers (n = 9), 53.5% for intermediate metabolizers (n = 8) and 17.9% for PMs (n = 4), *P* = 0.230). Therefore, the differences in the plasma concentrations of regorafenib and its metabolites between ALBI grade groups do not seem to be caused by dose reductions necessitated by hyperbilirubinemia caused by inhibition of UGT1A1 by regorafenib; instead, they may be due to the continuation of a low maintenance dose that is the same as the low initial dose used for patients classified as ALBI grade 2.

Thus, we conclude that patients in the present study with ALBI grade 2 had lower plasma concentrations of regorafenib and its metabolites M-2 and M-5 due to their older age and lower doses. In clinical practice, older patients or those who are classified as ALBI grade 2 or 3 are often treated with reduced doses of anticancer drugs in order to limit the occurrence of adverse events. The results of our present study suggest that when low doses of regorafenib are administered, plasma concentrations of the drug and its metabolites might not be maintained within a sufficient range. Because regorafenib is administered with a low initial dose followed by low maintenance doses, the plasma concentrations of regorafenib also remain low, which may reduce therapeutic effectiveness and shorten survival time. Therefore, regorafenib therapy might need to include close monitoring of plasma concentrations of the drug and its metabolites early during the first cycle to guide dose adjustments. Specifically, CRC patients often receive regorafenib during a course of treatments with other anticancer drugs, and their physical strength may be decreased. Therefore, clinicians must take into account the patient’s overall condition in addition to the ALBI grade. For CRC patients with ALBI grade 2 or 3, following administration of a relatively low initial dose (120 mg or less), the monitoring of plasma concentrations may indicate a need to gradually increase the dose.　On the other hand, for patients with advanced GIST and HCC refractory to prior chemotherapy, the administration of a relatively high initial dose of 160 mg may be more suitable, and this dose might be reduced if needed according to monitoring of plasma concentrations. However, only four patients with GIST and two patients with HCC were included in the present study. Therefore, further studies involving patients with GIST or HCC are warranted.

## Conclusion

The plasma concentrations of regorafenib and its major metabolites M-2 and M-5 were significantly lower in patients with ALBI grade 2 compared with patients with ALBI grade 1. Among patients with CRC who were receiving regorafenib therapy, ALBI grade 2 was associated with significantly shorter overall survival time than was ALBI grade 1. The administration of low-dose regorafenib to patients with ALBI grade 2 or 3 also seemed to reduce the RDI of regorafenib. Consequently, the treatment effect in these patients was reduced because appropriate plasma concentrations of regorafenib were not attained. These results emphasize the importance of plasma concentration monitoring and dose adjustments during regorafenib therapy.

## Data Availability

The data supporting the findings of this study are available within the article.
